# Exosomes derived from miR-188-3p-modified adipose-derived mesenchymal stem cells protect Parkinson’s disease

**DOI:** 10.1016/j.omtn.2021.01.022

**Published:** 2021-01-26

**Authors:** Qiang Li, Zihao Wang, Hao Xing, Yu Wang, Yi Guo

**Affiliations:** 1 Department of Neurosurgery, Peking Union Medical College Hospital, Chinese Academy of Medical Sciences and Peking Union Medical College, Beijing, China; 2 The First Affiliated Hospital of Bengbu Medical College, Anhui, China

**Keywords:** exosomes, Parkinson’s disease, adipose-derived mesenchymal stem cells, miR-188-3p, autophagy

## Abstract

Parkinson’s disease (PD) is the second-most common neurodegenerative disease after Alzheimer’s disease. The most important pathological feature of PD is the irreversible damage of dopamine neurons, which is related to autophagy and neuroinflammation in the substantia nigra. Previous studies found that the activation of NAcht Leucine-rich repeat Protein 3 (NLRP3) inflammasome/pyroptosis and cell division protein kinase 5 (CDK5)-mediated autophagy played an important role in PD. Bioinformatics analyses further predicted that microRNA (miR)-188-3p potentially targets NLRP3 and CDK5. Adipose-derived stem cell (ADSC)-derived exosomes were found to be excellent vectors for genetic therapy. We assessed the levels of injury, autophagy, and inflammasomes in 1-methyl-4-phenyl-1,2,4,5-tetrahydropyridine (MPTP)-induced PD mice models and neurotoxin 1-methyl-4-phenylpyridinium (MPP+)-induced cell models after treating them with miR-188-3p-enriched exosomes. miR-188-3p-enriched exosome treatment suppressed autophagy and pyroptosis, whereas increased proliferation via targeting CDK5 and NLRP3 in mice and MN9D cells. It was revealed that mir-188-3p could be a new therapeutic target for curing PD patients.

## Graphical Abstract

**Figure:**
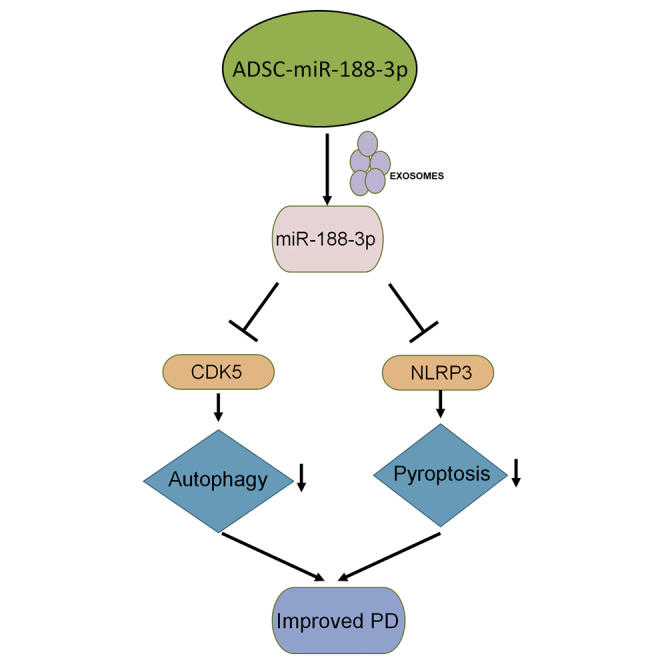

## Teaser

Guo and colleagues found that miR-188-3p is low expressed in the serum of PD patients. miR-188-3p-enriched exosomes derived from ADSCs ameliorated MPTP-induced Parkinson’s mice models and neurotoxin MPP+-induced cell models. miR-188-3p suppressed its target genes NLRP3 and CDK5, thereby inhibiting autophagy and pyroptosis both *in vitro* and *in vivo*.

## Introduction

Parkinson’s disease (PD) is a common chronic progressive neurodegenerative disorder that affects 2%–3% of the population ≥65 years of age and is more popular among those >80 years of age.^1^ The disability and reduced quality of life caused by PD have made it become a major and increasing burden on patients, families, careers, and healthcare systems.^2^ Recent studies have shown that adipose-derived stem cells (ADSCs) have therapeutic effects in several types of neurodegenerative disorders.^3^ More and more evidence indicated that exosomes secreted by ADSCs played an important role in ADSC therapy, and the implementation of exosomes derived from microRNAs (miRNAs)-overexpressed ADSCs provided protection in diseases.^4^, ^5^, ^6^ Exosomes are membrane vesicles that are secreted by most cells. Exosomes of 30−100 nm in diameter contain many macromolecular components, including proteins, mRNAs, and miRNAs, which can regulate intracellular signaling pathways.^7^ It is therefore important and promising to study miRNA-overexpressed, ADSC-derived exosome treatment in PD.

**Table 1:** Demographic characteristics of cases and controls (n = 20)

Characteristics	PD cases (n = 20)	Controls (n = 20)	p value
Age, mean ± SD (years)	70.1 ± 8.2	68.9 ± 8.1	>0.05

Gender, n			

Men	11	13	>0.05
Women	9	7	>0.05
Diabetes, n	8	9	>0.05
Hyperhomocysteinemia, n	11	11	>0.05
Cerebrovascular factors, n	10	13	>0.05
Hypertension, n	12	11	>0.05
Drinking, n	6	9	>0.05
Smoking, n	6	7	>0.05

A variety of factors are involved in the initiation and development of PD. Attention has recently focused on the contribution of autophagy and the inflammation response to the pathogenesis of PD.^8^, ^9^, ^10^ Autophagy and inflammasome were activated during the progress of PD, and an effective control of the activation of autophagy and inflammasome was expected to be a new therapeutic target for PD.^11^, ^12^, ^13^ NALP3 and cell division protein kinase 5 (CDK5) were found to, respectively, participate in the regulation of autophagy and inflammasome in PD.^11^^,^^14^ Bioinformatics analyses (https://www.genecards.org/) further predicted that microRNA (miR)-188-3p potentially targeted the 3′ UTR of NALP3 and CDK5 and mediated translational repression. Furthermore, a study has revealed that the expression of miR-188-3p was significantly decreased in ischemia reperfusion brain injury.^15^ miR-188-3p was also reported to bring an effective suppression on autophagy and apoptosis in myocardial infarction by targeting ATG7.^16^ Yet, the potential role of miR-188-3p in treating PD is still unknown in any research yet.

In our research, the level of miR-188-3p in PD patients was measured. We assessed the levels of injury, autophagy, and inflammation factors in MPTP-induced PD mice models and neurotoxin MPP+-induced cell models after treating them with miR-188-3p-enriched ADSC-derived exosomes. Further research confirmed that NALP3 and CDK5 are both direct targets of miR-188-3p. The data suggested that miR-188-3p-overexpressed exosomes had therapeutic effects on PD by suppressing the expression of autophagy and inflammasomes via targeting NALP3 and CDK5, both in the cell model and in the mice model.

## Results

### The injection of miR-188-3p-enriched exosomes alleviated substantia nigra damage in MPTP-induced mice models

**Figure 1:**
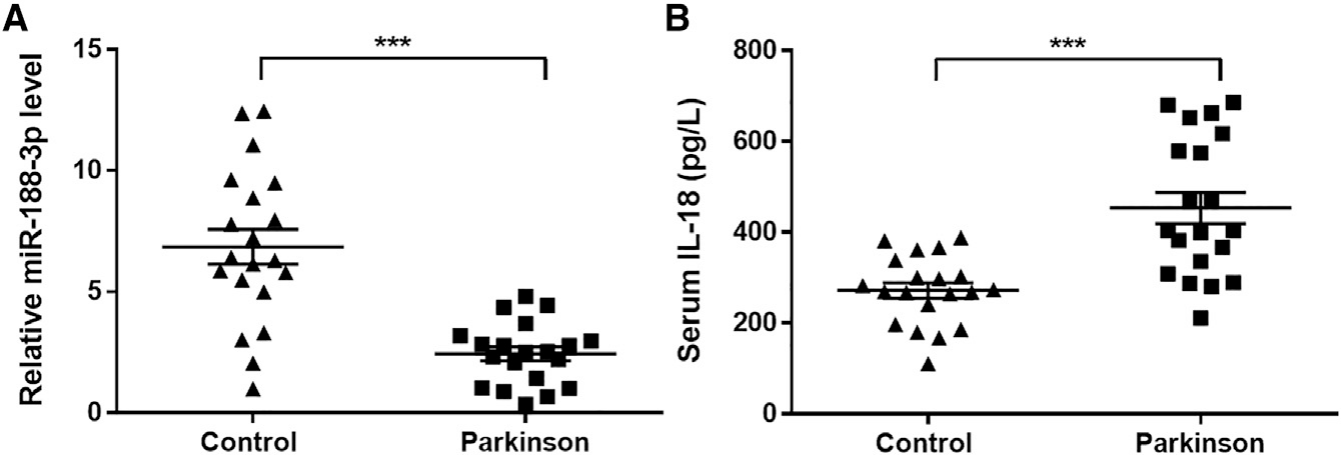
The level of miR-188-3p and IL-18 in Parkinson’s disease (PD) patients (A) RT-PCR results of miR-188-3p in healthy and PD patients. (B) The level of IL-18 in serum of healthy and PD patients. Data are expressed as mean ± SD (n = 20). ∗∗∗p < 0.001 compared to control.

**Figure 2:**
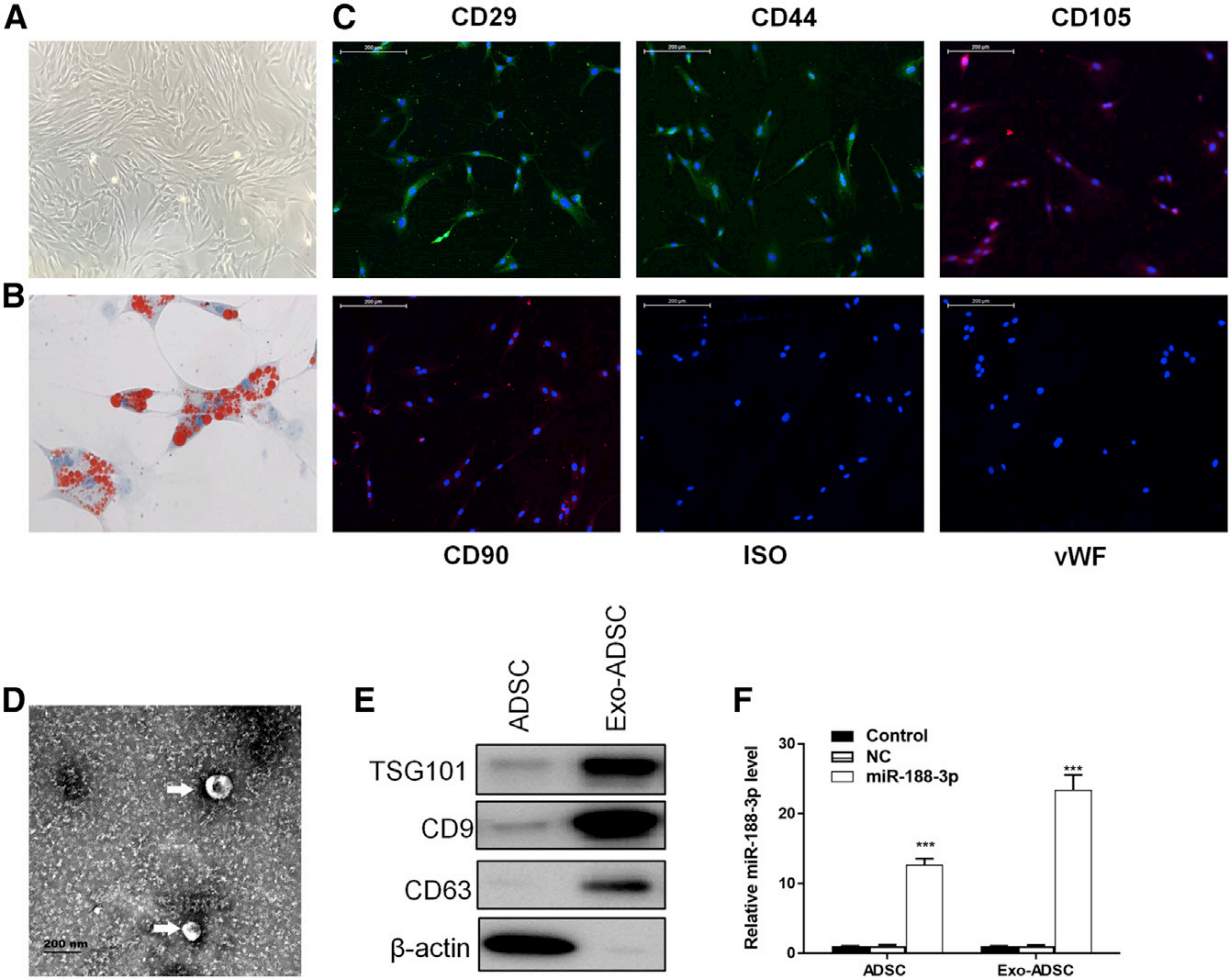
The confirmation of miR-188-3p-enriched ADSC-derived exosomes (A) ADSCs derived from C57 mice displayed a typical cobblestone-like morphology under a microscope. (B) Adipose cells were verified by oil red O staining. (C) ADSCs were positive for the MSC markers CD29, CD44, CD90, and CD105. (D) ADSC-derived exosomes were photographed under TEM. (E) Western blotting results of ADSC and ADSC-derived exosomes. (F) RT-PCR results of miR-188-3p in ADSC and ADSC-derived exosomes in control groups and miR-188-3p-overexpression groups. Data are reported as mean ± SD, n = 3. ∗∗∗p < 0.001.

The serum from healthy people and PD patients was collected and analyzed. RT-PCR revealed that the miR-188-3p level in PD patients was extremely lower (Figure 1A). Interleukin-18 (IL-18) was recognized as an important proinflammatory cytokine.^17^ Enzyme-linked immunosorbent assay (ELISA) showed that IL-18 in serum of PD patients increased significantly (Figure 2B). The demographic characteristics of cases was displayed in Table 1.

ADSCs were derived from C57 mice and displayed a typical cobblestone-like morphology (Figure 2A). Adipose cells were verified by Oil Red O staining (Figure 2B). Results showed that ADSCs were positive for the mesenchymal stem cell (MSC) markers CD29, CD44, CD90, and CD105 (Figure 2C). The exosomes of 50−120 nm in diameter secreted by ADSCs were then photographed using transmission electron microscopy (TEM) (Figure 2D). TSG101, CD9, and CD63 were typical surface markers of exosomes.^18^ Western blotting results confirmed the derived exosomes (Figure 2E). The ADSCs were transfected for miR-188-3p overexpression. RT-PCR analyses showed that the transfection and expression were effective in ADSCs, and the expression of miR-188-3p was increased in both ADSCs and the derived exosomes (Figure 2F).

**Figure 3:**
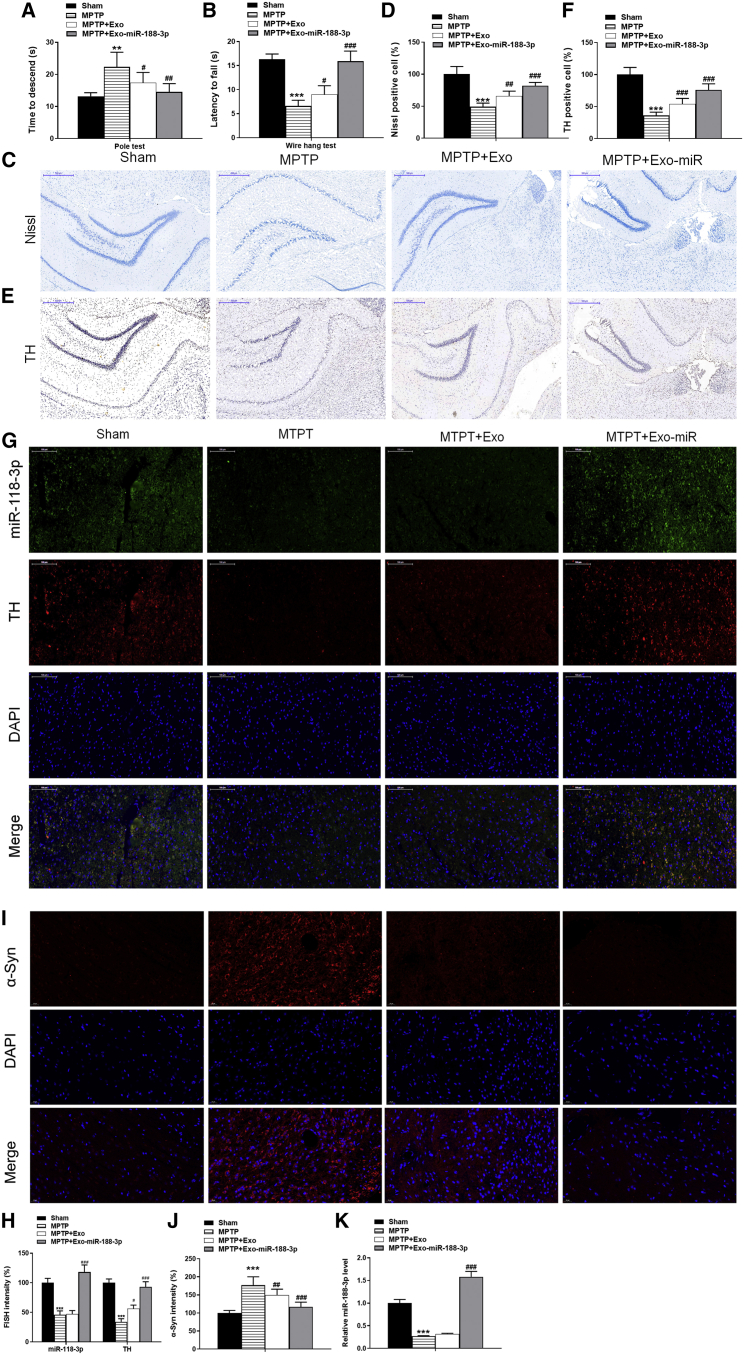
miR-188-3p-enriched exosomes alleviated substantia nigra damage in MPTP-induced mice models We constructed MPTP-induced mice models and injected them with miR-188-3p-enriched exosomes. (A) Mice pole test results. (B) Mice wire-hang test results. (C) Nissl staining results of mice in sham, MPTP, MPTP + normal exosomes, and MPTP + miR-188-3p-enriched exosome groups. (D) Quantitative analyses of Nissl staining results. (E) Immunohistochemistry results of TH in sham, MPTP, MPTP + normal exosomes, and MPTP + miR-188-3p-enriched exosome groups. (F) Quantitative analyses of immunohistochemistry results. (G) FISH results of miR-188-3p and TH in sham, MPTP, MPTP + normal exosomes, and MPTP + miR-188-3p-enriched exosome groups. (H) Immunofluorescence results of α-syn in sham, MPTP, MPTP + normal exosomes, and MPTP + miR-188-3p-enriched exosome groups. (I) Quantitative analyses of FISH intensity. (J) Quantitative analyses of immunofluorescence intensity. (K) RT-PCR results of miR-188-3p in sham, MPTP, MPTP + normal exosomes, and MPTP + miR-188-3p-enriched exosome groups in mice SNpc. Data are reported as mean ± SD, n = 8. ∗p < 0.1, ∗∗p < 0.01, ∗∗∗p < 0.001.

**Figure 4:**
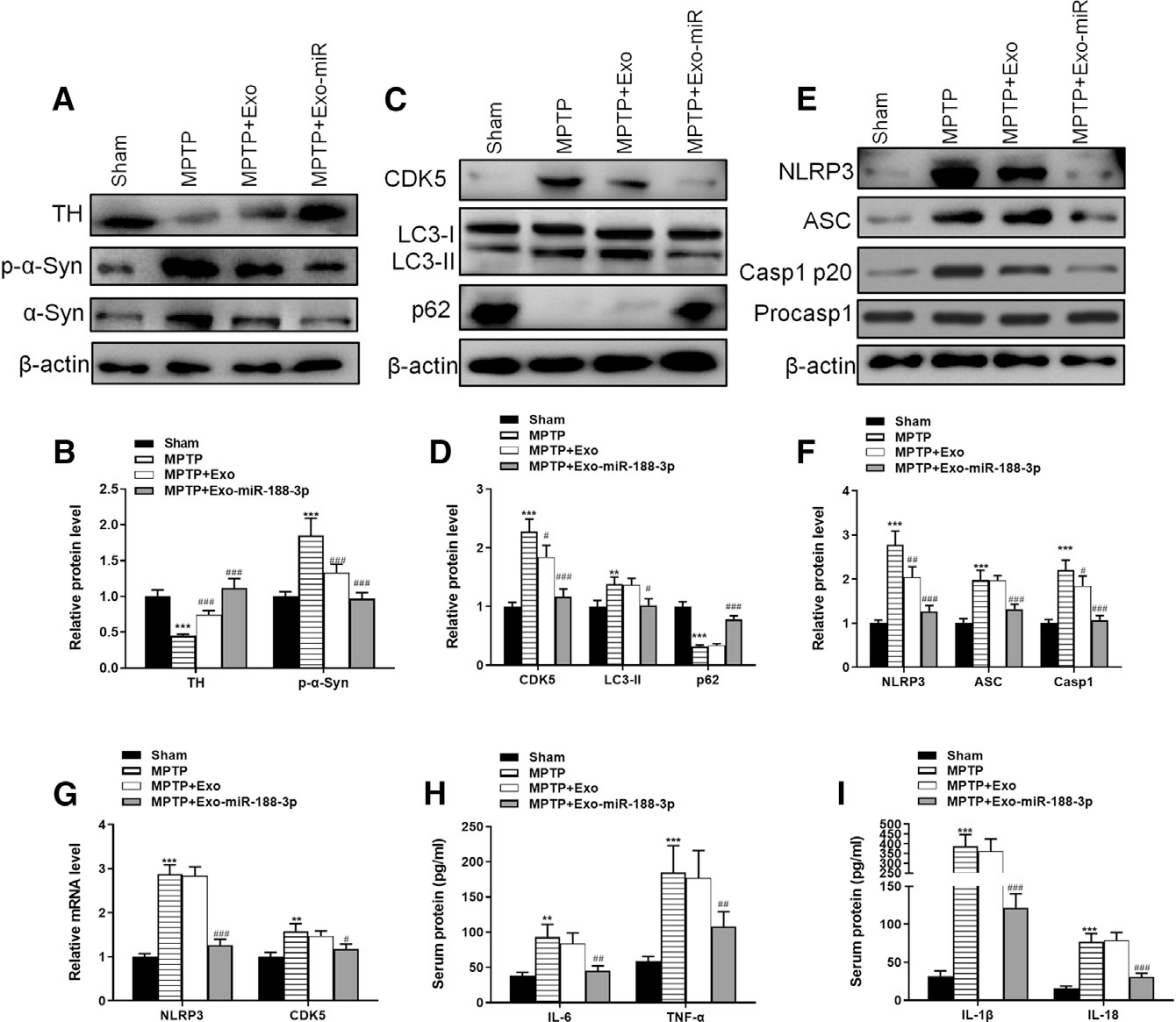
The injection of miR-188-3p-enriched exosomes suppressed the levels of CDK5 and NLRP3 in MPTP-induced PD mice (A) Western blot results of TH, α-syn, and p-α-syn in sham, MPTP, MPTP + normal exosomes, and MPTP + miR-188-3p-enriched exosome groups in mice SNpc. (B) Quantitative analyses of western blot results. (C) Western blot results of CDK5, LC3-I, LC3-II, and p62 in each group in mice SNpc. (D) Quantitative analyses of western blot results. (E) Western blot results of NLRP3, ASC, Casp1 p20, and Procasp1 in each group in mice SNpc. (F) Quantitative analyses of western blot results. (G) RT-PCR results of NLRP3 and CDK5 in each group. (H) The levels of IL-6 and TNF-α in serum from each group. (I) The levels of IL-1β and IL-18 in serum from each group. Data are reported as mean ± SD, n = 8. ∗p < 0.1, ∗∗p < 0.01, ∗∗∗p < 0.001.

PD’s most important pathogenesis is associated with damage to substantia nigra neurons.^19^^,^^20^ The mice models were constructed by inducing them with MPTP and were then injected with miR-188-3p-enriched exosomes. To verify the effects of miR-188-3p-overexpressed exosomes on behavioral alterations, we performed a pole test and wire-hang test. As shown in Figure 3A, with miR-188-3p-enriched exosome treatment, the latency to reach the floor was significantly reduced. In the wire-hang test, MPTP reduced the hanging time, indicating that the neuromuscular strength was significantly compromised while this effect was abrogated with miR-188-3p-overexpressed exosome treatment (Figure 3B). MPTP treatment in mice is known to severely damage dopaminergic (DAergic) terminals in the striatum-producing losses in catecholamines, which are linked to acute changes in locomotor behavior.^21^ Hence, in our further study, we will focus the alteration of catecholamine levels in order to better explain the effect of miR-188-3p-enriched exosomes on mice locomotor behavior. The Nissl staining was used for assessment of the damage to neurons in substantia nigra via staining Nissl bodies. Results indicated that MPTP inducement brought injury to substantia nigra, and general exosome treatment slightly increased the percentage of Nissl-positive cells, whereas miR-188-3p-overexpressed exosomes significantly repaired substantia nigra damage (Figures 3C and 3D). Tyrosine hydroxylase (TH) was widely considered an indicator of PD in substantia nigra.^22^ Immunohistochemistry was used to analyze the expression of TH proteins, and results showed that the expression of TH in the MPTP group was extremely reduced, and injection with general exosomes brought a little increase, whereas that with miR-188-3p-overexpressed exosomes brought a much larger increase to the expression of TH (Figures 3E and 3F). Fluorescence *in situ* hybridization (FISH) and western blotting analysis further confirmed the effect of miR-188-3p-enriched exosomes on TH in MPTP-induced mice (Figures 3G, 3H, 4A, and 4B). α-synuclein (α-syn) has been identified as a pathological hallmark of PD.^23^ Our Immunofluorescence and western blotting results demonstrated that MPTP induced the expression of α-syn, and miR-188-3p-enriched exosomes significantly abrogated the level of α-syn in substantia nigra pars compacta (SNpc) (Figures 3I, 3J, 4A, and 4B). RT-PCR analysis showed that in the PD mice group, the expression of miR-188-3p was suppressed distinctly, and the expression rose higher after injection with miR-188-3p-enriched exosomes than that before MPTP inducement in mice SNpc (Figure 3K). These results combined indicated that the injection of miR-188-3p-enriched exosomes alleviated substantia nigra damage in MPTP-induced mice models.

### The injection of miR-188-3p-enriched exosomes suppressed the levels of CDK5 and NLRP3 in MPTP-induced PD mice

The expression of CDK5 and autophagy pathway trackers LC3 and p62 in mice SNpc was measured with western blotting. Results showed that MPTP inducement increased the level of CDK5 and LC3-II/I by 2.3- and 1.4-fold, whereas reduced the level of p62 protein by 0.32-fold. General exosome injection had little effect in the mice, but exosomes with miR-188-3p overexpression reversed the effects of MPTP on CDK5, LC3-II/I, and p62 by 0.5- and 2.3-fold (Figures 4C and 4D). We used similar methods to study the expression level of NLRP3 and that of Casp1 p20, which was related to pathway of pyroptosis. Results showed that in the MPTP group, NLRP3, ASC, and Casp1 p20 were increased significantly by 2.8-, 2.0-, and 2.2-fold, respectively; miR-188-3p-enriched exosome treatment suppressed the expression of them by 0.7-, 0.66-, and 0.48-fold, respectively (Figures 4E and 4F). RT-PCR confirmed the expression of CDK5 and NLRP3 in all four groups (Figure 4G).

To assess the level to inflammasome and pyroptosis, serum of mice was collected to be analyzed using ELISA. The levels of inflammatory factors IL-6 and tumor necrosis factor (TNF)-α revealed that MPTP enhanced inflammation, and miR-188-5p restrained the inflammation level (Figure 4H). ELISA analysis of factors IL-1β and IL-18 also confirmed that the pyroptosis level was extremely higher after MPTP-inducement, whereas miR-188-5p treatment suppressed pyroptosis effectively (Figure 4I). These results suggested that miR-188-3p-enriched exosomes suppressed the levels of CDK5, NLRP3, autophagosome accumulation, and pyroptosis in MPTP-induced PD mice.

### miR-188-3p-enriched exosome treatment suppressed autophagy and pyroptosis, whereas increased proliferation via targeting CDK5 and NLRP3 in MPP+-induced MN9D cells

**Figure 5:**
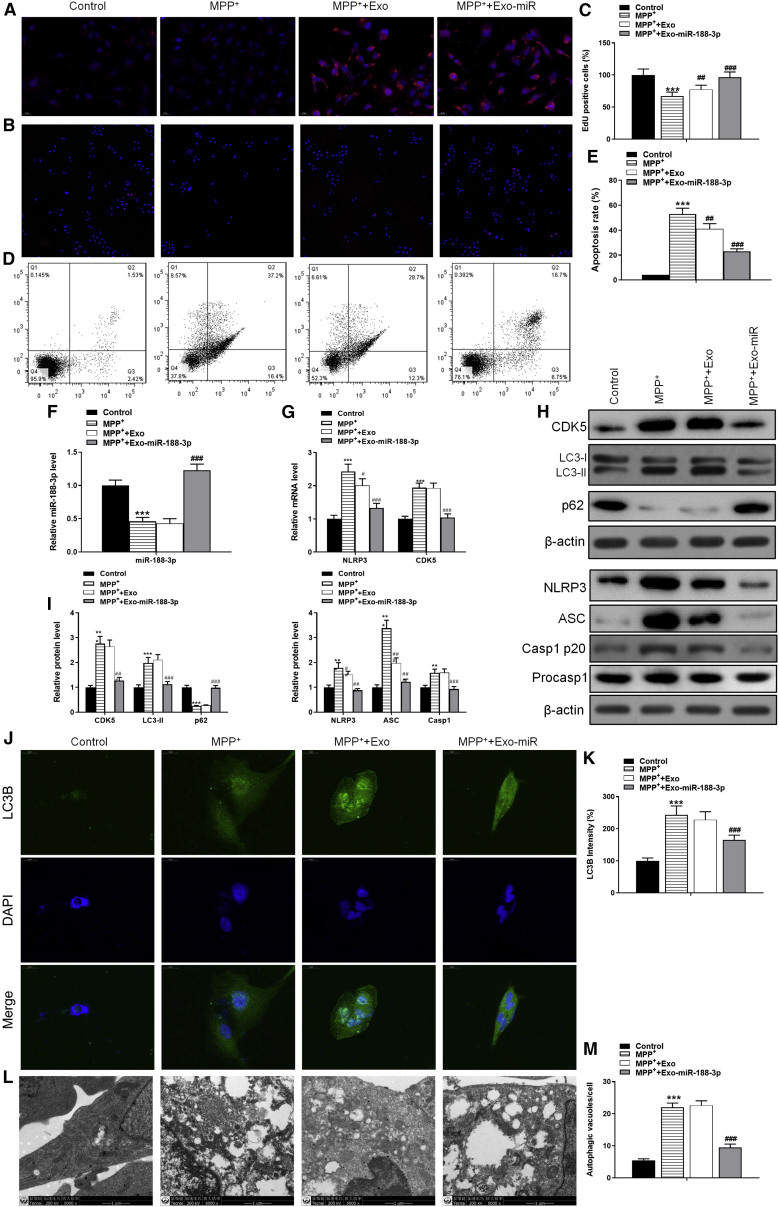
miR-188-3p-enriched exosome treatment suppressed autophagy and pyroptosis, whereas increased proliferation in MPP+-induced MN9D cells PD cell models were constructed through using MPP+ to induce MN9D mouse cells. (A) Immunofluorescence of Dil (red)-labeled exosomes. (B) EdU assay results of proliferation level in control, MPP+, MPP+ with normal exosomes, and MPP+ with miR-188-3p-enriched exosome groups. (C) Quantitative analyses of the Edu assay. (D and E) Flow cytometry results of the apoptosis rate in MN9D cells. (F) RT-PCR results of miR-188-3p in each group. (G) RT-PCR results of NLRP3 and CDK5 in each group. (H) Western blot results of CDK5, LC3-I, LC3-II, and p62 in each group. (I) Quantitative analyses of western blot results. (J) Immunofluorescence intensity of LC3B in each group. (K) Quantitative analysis of immunofluorescence results. (L and M) Photographs by TEM to identify autophagy in each group and quantified. Data are reported as mean ± SD, n = 3. ∗p < 0.1, ∗∗p < 0.01, ∗∗∗p < 0.001.

*In vitro* PD models were constructed through using MPP+ to induce MN9D mouse cells. ADSC-derived exosomes labeled with Dil were cocultured with MN9D cells (Figure 5A). The proliferation level was measured using a 5-ethynyl-2′-deoxyuridine (EdU) assay. It was clear that MPP+ caused a fall of red puncta, as being cultured with general exosomes has no significant effects on the red puncta; nevertheless, miR-188-3p-overexpressed exosomes could extremely enhance proliferation (Figures 5B and 5C). Annexin V staining, followed by flow analysis, showed increased apoptosis in MPP+ cells, while this effect was abrogated with miR-188-3p-overexpressed exosomes (Figures 5D and 5E). RT-PCR detection provided proof that culturing cells with miR-188-3p-overexpressed exosomes successfully gave a rise to the expression of miR-188-3p, which directly caused a significant effect on suppressing NLRP3 and CDK5 (Figures 5F and 5G). Furthermore, western blotting tests confirmed the suppression of miR-188-3p on NLRP3 and CDK5 (Figures 5H and 5I). The levels of autophagy-related factors LC3-II/I and p62 and pyroptosis-related factors Casp1 p20 *in vitro* were also analyzed via western blotting methods. Results showed that the expressions of LC3-II/I and Casp1 p20 were increased after MPP+ inducement by 1.9- and 1.6-fold and were then decreased after being cultured with miR-188-3p-enriched exosomes by 0.56- and 0.59-fold (Figures 5H and 5I).

Immunofluorescence technique was brought in to verify the suppressing influence of miR-188-3p on an autophagy pathway. Staining results showed that the intensity of LC3B, which was strongly related to autophagy, was much higher in the MPP+ group. miR-188-3p-enriched exosomes significantly inhibited the expression compared to normal exosomes (Figures 5J and 5K). At the cellular level, photographs by TEM and following quantitative analysis confirmed that autophagy was suppressed (Figures 5L and 5M). These results revealed that miR-188-3p-enriched exosome treatment suppressed autophagy and pyroptosis in MPP+-induced MN9D cells.

### miR-188-3p directly targets the 3′ UTR NLRP3 and CDK5

**Figure 6:**
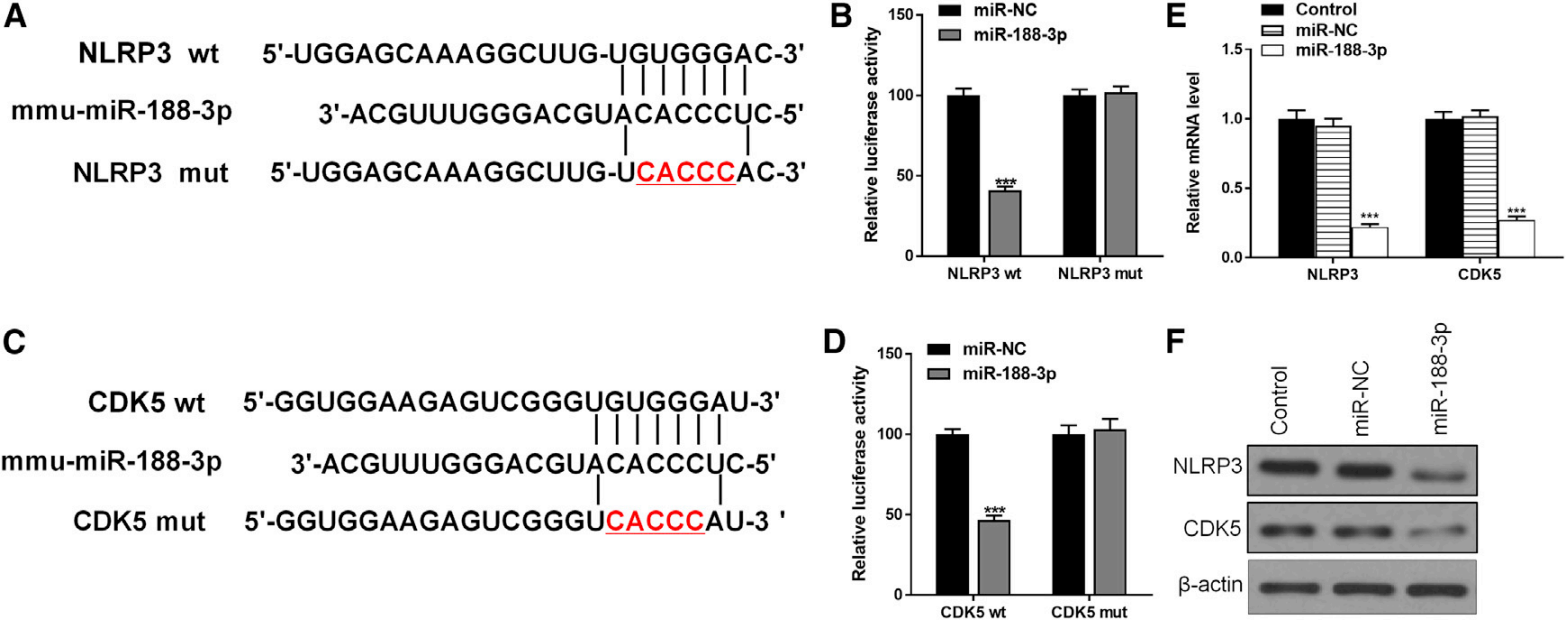
miR-188-3p directly targets the 3′ UTR NLRP3 and CDK5 To identify the potential role of miR-188-3p in PD, bioinformatics analyses were used to predict the possible targets. (A) Bioinformatics analyses show the connections between miR-188-3p and NLRP3. (B) Dual-luciferase assay results in NLRP3 WT and NLRP3 MUT groups. (C) Bioinformatics analyses show the connections between miR-188-3p and CDK5. (D) Dual-luciferase assay results in CDK5 WT and CDK5 MUT groups. (F) Western blot results of NLRP3 and CDK5in each group.Data are reported as mean ± SD, n = 3. ∗∗∗p < 0.001.

To identify the potential role of miR-188-3p in PD, bioinformatics analyses were used to predict the possible targets. Data showed that both NLRP3 and CDK5 could be the targets of miR-188-3p (Figures 6A and 6C). Dual-luciferase reporter assay results showed that overexpression of miR-188-3p reduced the intensity of fluorescence in HEK239T cells transfected with either a NLRP3-wild-type (WT) vector or CDK5-WT vector but had no effect on either a NLRP3-mutant (MUT) vector- or NLRP3-MUT vector-related intensity (Figures 6B and 6D). The results were similar in both mRNA and protein levels (Figures 6E and 6F).

**Figure 7:**
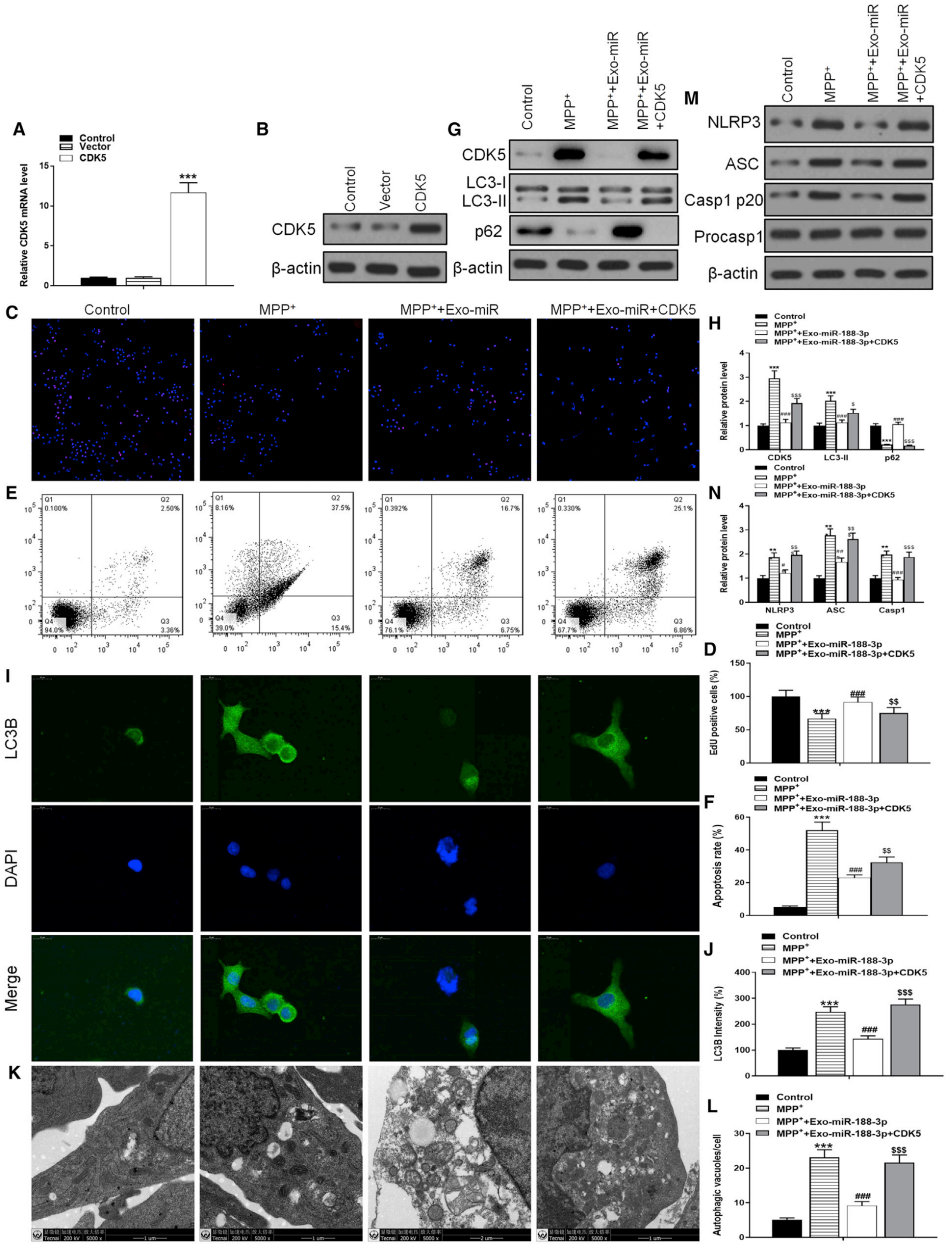
CDK5 overexpression inhibited the therapeutic effects of miR-188-3p-enriched exosomes (A) RT-PCR results of CDK5 in control, vector, and CDK5-overexpression groups. (B) Western blot results of CDK5 in each group. (C) EdU assay results in control, MPP+, MPP+ with miR-188-3p-enriched exosomes, and MPP+ with miR-188-3p exosomes + CDK5-overexpressed vectors groups. (D) Quantitative analyses of an EdU assay. (E and F) Flow cytometry results of the apoptosis rate in MN9D cells. (G) Western blot results of CDK5, LC3-I, LC3-II, and p62 in each group. (H) Quantitative analyses of western blot results. (I) Immunofluorescence analysis for LC3B in each group. (J) Quantitative analyses for immunofluorescence results. (K) TEM photographs verified the level of autophagy. (L) Quantitative analyses of TEM photos. (M) Western blot results of NLRP3, ASC, Casp1 p20, and Procasp1 in each group. (N) Quantitative analyses of western blot results. Data are reported as mean ± SD, n = 3. ∗∗∗p < 0.001.

To further confirm that miR-188-3p directly targets CDK5 and NLRP3, both two kinds of overexpressed vectors were constructed and transfected into cells. After being transfected, we detected the transfection efficiency in mRNA and protein levels using RT-PCR and western blotting, respectively (Figures 7A and 7B). The EdU assay showed that the cell proliferation, which was enhanced by miR-188-3p, would decrease to a level similar with that in the MPP+-inducement group after the cells were transfected with CDK5 (Figures 7C and 7D). Flow cytometry results indicated that overexpression of CDK5 reversed the anti-apoptotic effect of miR-188-3p-enriched exosomes (Figures 7E and 7F). Immunofluorescence analysis provided a same result about autophagy, and transfection with CDK5 reversed the expression of autophagy-related LC3B after it was suppressed by miR-188-3p (Figures 7I and 7J). At the cellular level, TEM photographs verified that even though autophagy was restrained through miR-188-3p treatment, CDK5 transfection brought a huge increase of autophagy to the cells (Figures 7K and 7L). Noticeable results were provided by western blotting analyses (Figures 7G and 7H). CDK5 overexpression in cells reversed the miR-188-3p-caused fall of the NLRP3 level and inflammasomes ASC and Casp1 p20 by 1.61-, 1.57-, and 2.02-fold (Figures 7M and 7N). These results suggested that miR-188-3p inhibited the expression of inflammasomes through restraining autophagy accumulation.

**Figure 8:**
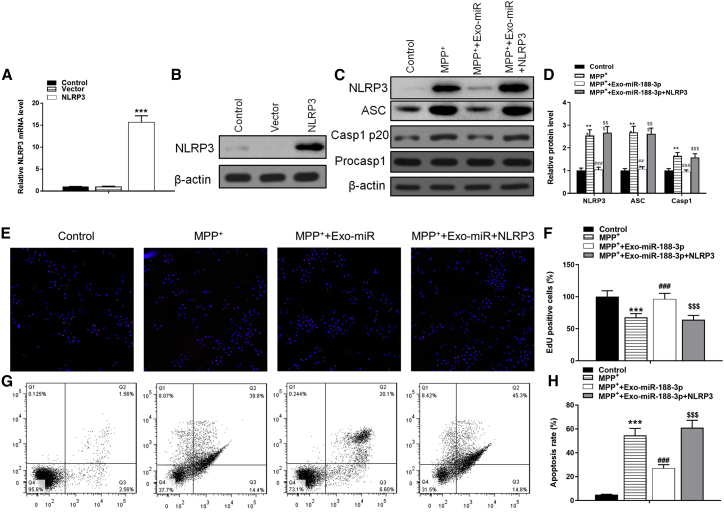
NLRP3 overexpression inhibited the therapeutic effects of miR-188-3p-enriched exosomes (A) RT-PCR results of NLRP3 in control, vector, and NLRP3-overexpression groups. (B) Western blot results of NLRP3 in each group. (C) Western blot results of NLRP3, ASC, Casp1 p20, and Procasp1 in each group. (D) Quantitative analyses of western blot results. (E) EdU assay results in each group. (F) Quantitative analyses of EdU assay. (G and H) Flow cytometry results of the apoptosis rate in MN9D cells. Data are reported as mean ± SD, n = 3. ∗∗∗p < 0.001.

As for NLRP3, the overexpressed vectors were transfected into MN9D cells with efficiency (Figures 8A and 8B). NLPR3 overexpression reversed the inhibitory effect of miR-188-3p on ASC and Casp1 p20 by 2.45- and 1.66-fold (Figures 8C and 8D). As a more noticeable proof about proliferation and apoptosis was discovered, NLRP3 transfection not only reversed the enhancing effect of miR-188-3p exosomes on proliferation and apoptosis but also brought the EdU-positive rate to a lower level than the MPP+-inducement group and apoptosis rate to a higher level (Figures 8E−8H).

Collectively, these results suggested that the therapeutic effects of miR-188-3p on PD mice and cells came from its ability to suppress autophagosomes and inflammasomes via targeting CDK5 and NLRP3. Overexpression of CDK5 and NLRP3 would erase the protective effects.

## Discussion

The pathogenesis of PD is very complex. Among all of the pathological features of PD, an important one is the irreversible damage of dopamine neurons in the substantia nigra.^24^ It has been revealed that neuroinflammation was present in the substantia nigra and facilitated the development of PD.^25^, ^26^, ^27^ The potential contribution of autophagy to the progress of neurodegenerative diseases has also been studied recently.^10^^,^^13^^,^^28^ Some studies presented direct evidence that the activation of NLRP3 inflammasome/pyroptosis and CDK5-mediated autophagy played an important role in PD.^11^^,^^14^^,^^29^ As ADSC-derived exosomes have been studied and applied in much research, miR-188-3p has been found to target ATG7 and inhibit autophagy and apoptosis during myocardial infarction.^16^ These findings became the basis of our research and inspired us with the design of experiments.

In this study, we confirmed that the expression of CDK5 and NLRP9 increased extremely in PD models, which led to autophagy, neuroinflammation, and apoptosis. We have shown that miR-188-3p directly targeted the 3′ UTR of NLRP3 and CDK5, and miR-188-3p-enriched exosomes suppressed autophagy and pyroptosis efficiently both in MPTP-induced mice and MPP+-stimulated MN9D cells. Moreover, we identified high cell proliferation after miR-188-3p-enriched exosome treatment *in vitro*. This is the first report on miR-188-3p having potential therapeutic effects on PD. In addition, we first used miRNA-enriched exosomes as a possible therapeutic tool in treating PD.

Autophagy is a vital part of our study. It has been shown in much research, including this study, that autophagy is involved in the pathogenesis of PD. However, little is known about the regulation of autophagy in neurodegenerative process. Activation of autophagy-induced DAergic neuron damage promotes removal of the forming cytotoxic proteins and structures.^30^ Su et al.^14^ have found that in MPTP-induced PD models of monkeys, the substantia nigra showed aggregation of α-syn and massive loss of DAergic neurons, as well as increased activity and expression of CDK5 and a large amount of autophagy. We also observed similar findings during this study, as well as the increased level of LC3-II. Interestingly, some studies showed that MPTP inhibited autophagy through the downregulation of LC3-II and the upregulation of p62, which is contrary to what we found.^31^ All of these studies implied an urgent direction of research, which is to decide the role of autophagic dysfunction in PD.

Exosomes played as intermediary agents in this study, whereas miR-188-3p is the leading study object during our experiments. We would like to assume that being enriched in exosomes and injected into mice models or cultured with MN9D cells is a better way for miR-188-3p to find its therapeutic effects and molecular mechanisms when comparing it with miR-188-3p-overexpressed lentiviral vectors. However, we did not find proofs beyond reasonable doubts that indicated miR-188-3p-enriched exosomes had better suppressing effects on autophagy and inflammasomes than miR-188-3p lentiviral vectors, *in vivo* or *in vitro*. In clinical applications, exosomes were expected to perform more efficiently and safely due to their compatibility with human bodies.

Thus, our data suggest that miR-188-3p inhibits autophagy and inflammasomes by targeting CDK5 and NLRP9 in the pathogenesis of PD. Treatment with miR-188-3p-enriched exosomes has a superb restoration effect on damage of neurons in the substantia nigra. We speculate that miR-188-3p could be a new therapeutic target for curing PD, and we expect miR-188-3p-enriched exosomes to become an effective treatment for PD patients.

## Materials and methods

### Animals

6-month C57BL/6J male mice were purchased from the Shanghai Laboratory Animal Center of the Chinese Academy of Sciences (Shanghai, China). Experimental animals were fed a standard laboratory diet and had *ad libitum* access to water. Mice were housed in a controlled environment with a temperature of 22°C ± 1°C, 65% ± 5% humidity, and a 12:12-h light/dark cycle. All animal experiments were conducted in accordance with the Institutional Guidelines for the Care and Use of Laboratory Animals of Peking Union Medical College Hospital.

### Adipose-derived mesenchymal stem cell culture

Adipose tissues obtained from euthanized C57BL/6 mice were rinsed in phosphate-buffered saline (PBS) and cut into 1 × 1 mm pieces. After digestion with collagenase, tissues were centrifuged at 4,000 × *g* for 5 min. The resultant cell pellet was then suspended in Dulbecco’s modified Eagle’s media (DMEM) containing 10% fetal bovine serum (FBS), 1% penicillin-streptomycin, and 2 mM L-glutamine. Cells were then cultured for 48 h in a controlled 38°C atmosphere with 5% CO_2_. Cells were transferred to fresh culture media every 3 days. When cells reached ~90% confluency, they were passaged and used at passage three. Cells were incubated with conjugated monoclonal antibodies against CD29, CD44, CD90, and CD105 to confirm the identity of ADSCs, whereas isotype-identical antibodies (Pharmingen, San Diego, CA, USA) served as controls. ADSCs were then fixed in 1% paraformaldehyde (PFA), and a FACSCalibur flow cytometer (BD Biosciences, San Jose, CA, USA) and FlowJo software (FlowJo, Ashland, OR, USA) were used for quantitative analyses. Logarithmic fluorescence intensities were recorded for 10,000–20,000 cells per sample.

### Isolation and analysis of exosomes

The negative control (NC) and miR-188-3p overexpression vectors (miR-188-3p mimic) were provided by GenePharma (Shanghai, China) and were transfected into ADSCs at a final concentration of 20 nM using Lipofectamine 3000 (Invitrogen Life Technologies, Carlsbad, CA, USA). ADSCs were collected for analysis of miR-188-3p levels at 48 h post-transfection. ADSCs (miR-188-3p mimic, control, and NC groups) at 80%–90% confluency were washed with PBS and cultured in microvascular endothelial cell growth media-2, free of FBS. ADSCs were then supplemented with 1 × serum replacement solution (PeproTech, Rocky Hill, NJ, USA) for 24 h. To remove dead cells and debris, ADSCs were centrifuged at 300 × *g* for 10 min, followed by 2,000 × *g* for 10 min, after which 5 mL of ExoQuick-TC reagent (System Biosciences, Palo Alto, CA, USA) was mixed with 10 mL of supernatant. After centrifugation at 1,500 × *g* for 30 min, the exosome-containing pellet was resuspended in nuclease-free water. TRIzol-LS (Invitrogen) and an Exosomal Protein Extraction Kit (Invitrogen) were used for extracting total RNA and protein, respectively. Exosomes were used immediately for experiments or stored at −180°C. A NanoSight LM10 (Malvern Instruments, Malvern, UK) nanoparticle tracking system was used to determine the sizes of purified exosomes. Western blotting was used to measure CD9, CD63, and TSG101 protein levels. To assess the protein concentration of exosomes, we used a bicinchoninic acid assay kit (Beyotime, Suzhou, China). TEM was performed on a Libra 120 (Zeiss, Oberkochen, Germany) to analyze vesicle ultrastructure.

### Animal experimentation

MPTP (30 mg/kg; Sigma-Aldrich, USA) was injected intraperitoneally in C57BL/6 mice (n = 8). MPTP was injected daily for 5 days. For the delivery of exosomes in MPTP mice, we isolated exosomes (400 μg of protein) in PBS and administered them by intravenous injection for 5 days. After completion of the treatment and behavior analysis, mice were anesthetized with isoflurane and transcardially perfused with ice-cold PBS. Blood was withdrawn after anesthetized. Blood samples were kept at room temperature for 2 h and then centrifuged at 840 × *g* for 10 min to collect serum. One-half of the SNpc was dissected and homogenized for western blot analysis. The other half of the SNpc was fixed in 4% PFA overnight at 4°C and incubated in 30% sucrose for immunostaining.

### Nissl staining

The midbrain tissues were fixed in 4% PFA, embedded in paraffin, and cut into 4 μm-thick sections. For Nissl staining, the sections were incubated with Nissl staining solution at 50°C for 20 min. After rinsing with distilled water, sections were dehydrated with 95% ethylalcohol and 70% ethyl alcohol in secession. The number of staining cells in SNpc was counted using a BX51 light microscope (Olympus, Tokyo, Japan) at higher magnification (×400).

### Cell culture

MN9D cells were purchased from Shanghai Institute of Cell Biology (Shanghai, China). Cells were cultured in DMEM high glucose medium (Gibco, Carlsbad, CA, USA) containing 10% FBS (Gibco, Carlsbad, CA, USA) and 100 U/mL of penicillin (Invitrogen) and 100 μg/mL of streptomycin (Invitrogen). The cells were incubated at 37°C in an incubator with a humidified atmosphere of 95% air and 5% CO_2_. MN9D cells were pretreated with 100 μM MPP+ (Sigma-Aldrich) for 24 h to establish the *in vitro* PD model.

### Luciferase reporter assay

To investigate whether miR-188-3p directly targeted CDK5 and NLRP3, the sequences of the 3′ UTR of CDK5 and NLRP3 were inserted downstream of a Renilla luciferase open-reading frame in the pGL3-CMV vector (Promega, Sunnyvale, CA, USA). We then transfected the MN9D cells with pGL3-basic along with an miR-188-3p mimic. After harvesting at 48 h, the luciferase activity was assessed. These experiments were performed at Yingbai (Shanghai, China).

### Statistical analysis

Data were expressed as mean ± standard error of the mean. The significance of differences between groups was evaluated by one-way ANOVA with a (LSD) post hoc test, and p < 0.05 was considered as statistically significant differences.

More details are in Supplemental materials and methods.

## Supplemental information

Document S1. Supplemental materials and methods

Document S2. Article plus supplemental information
