# Supplementary material for: Exosomes derived from miR-188-3p-modified adipose-derived mesenchymal stem cells protect Parkinson’s disease
Source: Mol Ther Nucleic Acids. 2021 Jan 26;23:1334–44. doi: 10.1016/j.omtn.2021.01.022 (PMC7920810; doi:10.1016/j.omtn.2021.01.022)
Supplement: Document S1. Supplemental materials and methods [file mmc1.pdf]

OMTN, Volume *23*

Supplemental information

Exosomes derived from miR-188-3p-modified adipose-derived mesenchymal stem cells protect Parkinson's disease

Qiang Li, Zihao Wang, Hao Xing, Yu Wang, and Yi Guo

Supplementary Methods

Patients and Clinical Characteristics

Twenty PD patients (9 females and 11 males; average age 70.1±8.2 years) were recruited between OCT 1, 2018, and Feb 31, 2019, at the Peking Union Medical College Hospital, Beijing, China. All patients were diagnosed as having PD according to the electronic computerized hospitalization records. Each participant provided signed informed consent prior to participate in the present study. Age- and sex-matched healthy individuals were also selected from the Peking Union Medical College Hospital (7 males and 13 females; average age 68.9±8.1 years). All serum samples were frozen in liquid nitrogen immediately after taken from the cubital vein within 24 hr of symptom onset and were used for further analysis. The clinical Characteristics were shown in Table.1.

Pole Test

The behavioral tests were performed after the completion of the treatment. Mice were positioned head up near the top of a rough-surfaced wooden pole (15 mm in diameter and 40 cm in height), and the time taken to reach the floor was considered. The test was repeated three times, and behavioral alterations were analyzed according to the mean of the three descending times.

Wire Hang Test

The neuromuscular strength was determined by the wire hang test. Before the test, the mice were acclimated to the behavioral room for 30 min. Mice were raised by their tail, placed smoothly on a horizontally stretched wire, and supported until they grabbed the wire with both of their hind and fore paws. The wire was mounted 20 cm above the ground surface to discourage falling but not cause any sort of injury in case of a fall. The experimental procedures were reiterated 10 times, and the average values were used in the evaluation. The mice were allowed to rest between the trials. The results are described in seconds, as a latency to fall to the ground.

Immunohistochemical analysis

Paraffin-embedded tissues (5 lm thickness) were firstly deparaffinized in xylene, and rehydrated in an ethanol gradient with distilled water. After endogenous peroxidase activity was quenched, liver sections were incubated with 5% bovine serum albumin to diminish nonspecific binding. After that, tissues were incubated with Tyrosine hydroxylase (TH) antibody at 4 °C overnight, and further detected using DAKO EnVision™ detection kits. All sections were counterstained with haematoxylin. The images were taken using an inverted microscope under the magnification of 200×.

Enzyme-linked immunosorbent assay

The concentration of IL-1β, IL-18, IL-6 and TNF-α in serum were determined using an ELISA kit (Nanjing Jiancheng Bioengineering Institute, Jiangsu Sheng, China) according to standard protocols.

Cell Transfection

To assess miR-188-3p expression, an miR-188-3p overexpression vector (miR-188-3p mimic) and negative control (miR-NC) were purchased from GenePharma. Cells were then transfected with either the miR-188-3p overexpression construct or miR-NC at a concentration of 50 nM using Lipofectamine 2000 (Invitrogen). Primary astrocytes were used for miR-188-3p expression analysis, or other experiments, at 48 h post-transfection.

To assess CDK5 and NLRP3 expression, CDK5 and NLRP3 overexpression vector constructs, and corresponding negative control constructs (Vector), were obtained from GenePharma. Cells were transfected with the CDK5 and NLRP3 overexpression vector at a final concentration of 50 nM, using Lipofectamine 2000 (Invitrogen), according to the manufacturer’s protocol.

RT-PCR analysis

RNA was isolated from tissues or cells using TRIzol reagent (Invitrogen). cDNA was synthesized from 1 μg of total RNA in a reaction volume of 21 μL, using oligo dT18 primers and SuperScript reverse transcriptase. PCR amplification was carried out with Taq DNA polymerase (TaKaRa, Tokyo, Japan) using 1 μL of the first-strand cDNA as template. The amplification reactions were run with 30 thermocycles of 30 s at 94°C, 30 s at 55°C and 30 s at 72°C. The expression levels were calculated using the $2^{\Delta\Delta CT}$ method[1].

Protein isolation and western blot analysis

Protein (50 μg) from lysed cells was separated by 10% SDS-PAGE, and transferred to nitrocellulose membranes, followed by blocking for 2 h. Next, membranes were incubated 2 hours at room temperature with primary antibodies, followed by horseradish peroxidase (HRP)-conjugated secondary antibodies[2]. The primary antibodies against TH (#58844, 1:1000), α-Synuclein (#4179, 1:1000), Phospho-α-Synuclein (Ser129) (#23706, 1:500), CDK5 (#14145, 1:1000), LC3B (#3868, 1:800), p62 (#23214, 1:1000), NLRP3 (#13158, 1:1000), ASC (#13833, 1:1000), Caspase-1 p20 (Cleaved Asp296) (#89332, 1:500), Caspase-1 (#2225, 1:1000) and β-actin (#4970, 1:5000). The antibodies were purchased from Cell Signaling Technology (Danvers, MA, USA). The protein bands were visualized using ECL Plus Detection Reagent (Applygen, Beijing, China).

EdU assay

The cell proliferation was detected using Cell-Light Apollo567 in vitro kit (RiboBio, Guangzhou, China). Cells were cultured in 1640 medium. EdU was applied at 20 μM. The cells were fixed with 4% paraformaldehyde and stained with Apollo 567 and Hoechst 33342.

Electron Microscopy

Cells were fixed with 2.5% glutaraldehyde in phosphate buffer and stored at 4°C until

embedding. Cells were then post-fixed with 1% osmium tetroxide followed by increasing dehydration gradients of ethanol and acetone. Cells were then embedded in Araldite, and ultrathin sections were obtained (50–60 nm). Sections were collected onto uncoated copper grids, and stained with 3% lead citrate-uranyl acetate. Images were examined with a CM-120 electron microscope (Philips).

[1] Huggett, J.; Dheda, K.; Bustin, S.; Zumla, A. Real-time RT-PCR normalisation; strategies and considerations, Genes Immun.6 (2005) 279-284, https://doi.org/10.1038/sj.gene.6364190.

[2] Mahmood, T.; Yang, P. C. Western blot: technique, theory, and trouble shooting, N. Am. J. Med. Sci.4 (2012) 429-434, https://doi.org/10.4103/1947-2714.100998.
